# Spectroscopic and Molecular Docking Studies on the Influence of Inulin on the Interaction of Sophoricoside with Whey Protein Concentrate

**DOI:** 10.3390/foods13223601

**Published:** 2024-11-11

**Authors:** Anna Wang, Mengyang Xie, Ligen Wu

**Affiliations:** 1 School of Food Science and Technology, Henan University of Technology, Zhengzhou 450001, China; wananna2006@163.com (A.W.); 13298179248@163.com (M.X.); 2 National Engineering Research Center of Wheat and Corn Further Processing, Henan University of Technology, Zhengzhou 450001, China

**Keywords:** sophoricoside, inulin, whey protein concentrate, FTIR, fluorescence spectrum, molecular docking

## Abstract

The influence of inulin on the interaction of sophoricoside (Sop) with whey protein concentrate (WPC) was investigated using various spectroscopic methods, including fluorescence spectroscopy (intrinsic fluorescence, synchronous fluorescence, and three-dimensional fluorescence), ultraviolet-visible (UV–Vis) spectroscopy, Fourier transform infrared (FTIR) spectroscopy, and molecular docking. Sop was found to quench the intrinsic fluorescence of WPC by a static mechanism, both with and without the addition of inulin, and to enhance the antioxidant capacity of the protein. The addition of inulin slightly increased the binding distance between WPC and Sop, while reducing the number of binding sites from two to one. Non-covalent interactions, predominantly van der Waals forces and hydrogen bonding, were maintained between Sop and the protein. Synchronous fluorescence spectroscopy revealed that Sop prevents the exposure of hydrophobic groups on tryptophan residues, leading to increased surface hydrophilicity of the WPC complex. This aligns with the decreased protein surface hydrophobicity measured by 8-Anilino-1-naphthalenesulfonic acid (ANS) binding assays. With inulin, the overall hydrophobicity of the protein was lower than in the system without inulin, suggesting that both inulin and Sop improve the solubility of WPC. Three-dimensional fluorescence spectral analysis showed a reduction in fluorescence intensity and a red shift in the presence of both Sop and inulin. FTIR spectroscopy indicated a slight increase in the secondary structure ordering of WPC following the addition of both Sop and inulin, suggesting structural stabilization under heating conditions. Molecular docking highlighted the potential for hydrogen bond formation between Sop and WPC.

## 1. Introduction

Whey protein concentrate (WPC), found primarily in animal milk [1], includes β-lactoglobulin (β-LG), α-lactalbumin (α-LA), bovine serum albumin (BSA), immunoglobulins, and other minor proteins [2]. Dairy proteins possess a high protein digestibility-corrected amino acid score (PDCAAS), indicating high bioavailability for digestion, absorption, and utilization [3]. WPC is involved in regulating human metabolism, playing a role in protein metabolism [4], and controlling blood sugar and lipid levels, which may influence weight management [5]. WPC isolate offers significant health benefits, including enhanced immune system function, antioxidant effects, anti-cancer properties, reduced fatigue, antibacterial activity, and bone strengthening [6]. The functionality of WPC can be altered through interactions with polysaccharides or small molecules, affecting its gelling capacity, interface activity [7], solubility, emulsification [8], thermal stability [9], and foaming properties. The antioxidant capacity [2] of WPC may be influenced by its interaction with inulin [10], while the observed reductions in appetite [11], energy intake (EI), and obesity [12] in some studies may also be attributed to this interaction. Consequently, there is considerable interest in enhancing the functional properties of proteins through modifications, such as conjugation with polyphenols or polysaccharides [13].

Inulin, a soluble dietary fiber also classified as a prebiotic, is extracted from chicory roots. The WCRF states that fiber offers protective effects against weight gain and obesity [12,14]. Research by Zeang Wu et al. [15] demonstrated that inulin has the potential to inhibit obesity in rats and has been used as a prebiotic to alleviate glucose and lipid metabolism disorders in mice and humans by modulating gut microbiota [16]. However, excessive consumption of inulin may lead to side effects, such as nausea, bloating, and flatulence [14].

**Figure 1:**
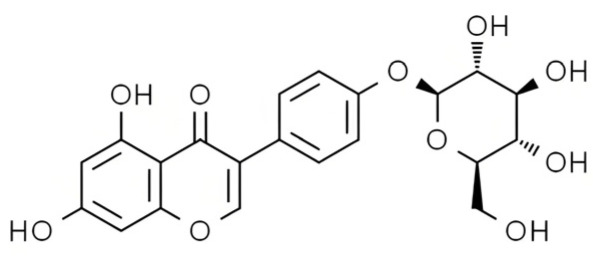
Sop Structure.

Phytochemicals, structurally diverse compounds naturally found in plants, exhibit anti-inflammatory [17], anticancer [18], and antioxidant properties. Research has shown that antioxidants can prevent the onset of neurodegenerative disorders, such as Alzheimer’s disease, Parkinson’s disease, and dementia [19]. Furthermore, phytochemicals have demonstrated specific effects in preventing diabetes, obesity, and cardiovascular diseases [20,21]. Sop (Figure 1), a principal component identified in the seed pod of the Chinese scholar tree, is classified as an isoflavone based on its chemical structure. Sop has shown estrogen-like effects, stimulating bone growth and showing potential for treating osteoporosis in postmenopausal women [22]. It has also been effective in treating dermatitis [23]. Sop has inhibited the proliferation of myocardial cells and aided in the regression of myocardial hypertrophy [24]. In experiments conducted on rats, Sop was found to suppress and treat persistent asthma [25], colitis [26], prostate hypertrophy [27], and liver injury [24]. As a specific inhibitor of LXRβ, Sop is under evaluation for its potential to alleviate fatty liver diseases and prevent complications, such as obesity and hyperlipidemia, arising from insulin-related disorders [28].

In this study, WPC was modified by adding Sop and inulin. The objective was to assess the impact of Sop on WPC, both before and after the incorporation of inulin, and to determine changes in quenching type, binding sites and distances, surface hydrophobicity, and secondary structure through the use of fluorescence spectroscopy, UV–Vis spectroscopy, FTIR spectroscopy, simulated molecular docking, and ANS.

## 2. Materials and Methods

### 2.1. Materials

Sop was obtained from Shanghai Yuanye Bio-Technology Co., Ltd. (Shanghai, China). WPC, inulin, and KBr were sourced from Shanghai Macklin Biochemical Technology Co., Ltd. (Shanghai, China); Na_2_HPO_4_ and NaH_2_PO_4_ were provided by Tianjin Kermel Chemical Reagent Co., Ltd. (Tianjin, China).

### 2.2. Sample Preparation

WPC was dissolved in a 0.1 M phosphate-buffered (PBS) solution at pH 9, stirred using a magnetic stirrer for one hour, and then allowed to rest overnight at 4 °C [29]. The final concentrations of WPC were 2% and 4%; inulin was present at a concentration of 4%. Sop was dissolved at 37 °C under magnetic stirring using 0.1 M PBS as the solvent, resulting in final concentrations of 120 μg/mL, 140 μg/mL, 160 μg/mL, 180 μg/mL, and 200 μg/mL, respectively.

Whey protein concentrate–sophoricoside complex (WPC–Sop) was prepared by mixing equal volumes of 2% WPC with different concentrations of Sop. The ternary complex (WPC–inulin–Sop) was formulated according to the method proposed by Wang Yu et al. [30] by mixing 4% inulin solution with WPC and different concentrations of Sop in the ratio of 1:1:2 by volume. After mixing, the samples were heated at temperatures of 363 K, 368 K, and 373 K for two hours. The samples were then cooled and centrifuged at 4 °C and 15,414× *g* for 10 min. Subsequently, the resulting supernatant was diluted sixfold to prepare the test solution. For the liquids to be measured, the number of measurements for each indicator was 3, and the final results were averaged.

### 2.3. UV–Vis Absorption Spectra Test Methods

The UV–Vis spectra measurements were performed using a UV7600 dual-beam UV–visible spectrophotometer (Shanghai Lengguang Technology Co., Ltd., Shanghai, China). The solution under examination was analyzed for UV–Vis absorption spectrum within the range of 260–450 nm, using 0.1 M PBS at pH 9 as a reference solution [31].

### 2.4. Fluorescence Spectroscopy

The fluorescence spectra of WPC–Sop and WPC–inulin–Sop were obtained using a fluorescence spectrometer (F98 Fluorospectrophotometer, Shanghai Lengguang Technology Co., Ltd., Shanghai, China). The method for fluorescence spectroscopy was adapted from the approach described by Chu Wenwen [32]. For burst fluorescence, the excitation and emission wavelengths were set at 280 nm and 310–500 nm, respectively. For synchronous fluorescence scans, the wavelength range was established from 250 to 360 nm with a wavelength shift Δλ of 15 nm (for tyrosine residues), and from 250 to 500 nm with a wavelength shift Δλ of 60 nm (for tryptophan residues). For the three-dimensional fluorescence spectrum, the excitation and emission wavelengths were defined within the range of 240–450 nm.

### 2.5. FTIR Measurement Test Methods

The initial step involved freeze-drying the sample. The freeze-dried samples to be tested were then mixed with dried KBr atis. Subsequently, the sample was combined with dried KBr in a 1:100 ratio, ground, and compressed into tablets [33]. The FT-IR analysis was performed using a Nicolet6700 Fourier infrared absorption spectrometer (Thermo Fisher Scientific (China), Shanghai, China) within the wavelength range of 4000–400 cm^−1^. The spectral resolution was set at 4 cm^−1^, and the 32 scans were conducted.

### 2.6. Molecular Docking Simulations Test Methods

The main components identified in WPC included α-La, β-Lg, BSA, and lactoferrin (LF), as determined from previous electrophoresis studies. The structures were retrieved from the PDB Protein Bank under the codes 1F6S, 1BEB, 4F5S, and 1B1X. Similarly, the structure of Sop was located in the Pubchem Chemical Database. Protein pKa values were predicted and structural modifications were made using PROPKA 3. The docking of proteins with Sop was simulated using AutoDockTools-1.5.7 software, with the docking process repeated 50 times. The optimal docking outcome was determined based on the lowest docking energy, and PyMOLWin-1.0.0.0 software was used for the visualization of the molecular docking details [34].

### 2.7. Surface Hydrophobicity Test Methods

The surface hydrophobicity of WPC was analyzed using ANS as a fluorescent probe [35]. A mixture of 30 µL of ANS solution at a concentration of 8 mmol/L and 4.8 mL of test solution was allowed to react for 30 min in the absence of light. A PBS solution served as the reference, and the fluorescence was measured with an emission wavelength of 467 nm and an excitation wavelength of 365 nm. The fluorescence spectrum of the test sample was recorded to assess the hydrophobicity of the WPC surface based on its fluorescence intensity.

## 3. Results

### 3.1. UV–Vis Absorption Spectra

Amino acids with aromatic properties, found in proteins, comprise three main components: tyrosine, tryptophan, and phenylalanine. The predominant absorption peak at approximately 280 nm in the ultraviolet absorption spectrum primarily results from the π→π* transition of these aromatic amino acids [36]. UV spectroscopy can be utilized to determine whether small molecules interact with proteins. If the absorbance at a wavelength of about 280 nm exhibits a significant change, it can be tentatively concluded that the two substances have formed a complex [37].

**Figure 2:**
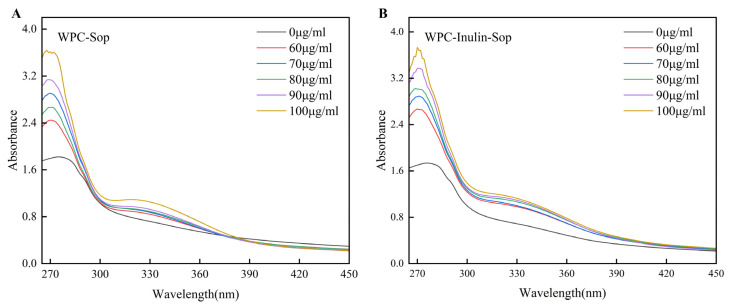
UV–Vis absorption spectra of the interaction between Sop and WPC, both before and after the addition of inulin. (**A**) depicts the UV–Vis absorption spectra of the WPC–Sop system, while (**B**) illustrates the UV–Vis absorption spectra of the WPC–inulin–Sop system.

Figure 2 illustrates the UV–Vis absorption spectra of the 373 K-treated WPC–Sop system and the WPC–inulin–Sop system. For the WPC–Sop system, the absorption peak of WPC at 270 nm showed a gradual increase and redshift with the increasing concentration of Sop. In the WPC–inulin–Sop system, the addition of inulin led to a slight increase in absorbance, indicating that inulin impacts the aromatic amino acids. The absorption spectrum at 270 nm exhibited a significant alteration, suggesting that Sop interacted with the surface of WPC [38].

### 3.2. Fluorescence Spectra

#### 3.2.1. Fluorescence Quenching Spectrum

**Figure 3:**
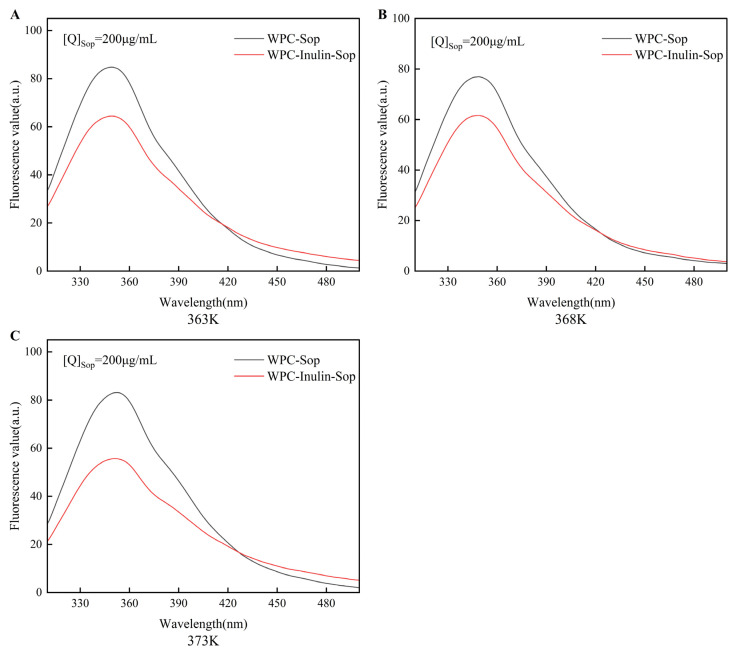
Fluorescence quenching spectra of the interaction between Sop and WPC before and after inulin addition. The interaction burst spectra of Sop–WPC and Sop–inulin–WPC at temperatures of 363 K, 368 K, and 373 K, respectively, are illustrated in (**A**–**C**).

As depicted in Figure 3, the fluorescence spectra were analyzed at an excitation wavelength of 280 nm (λex = 280 nm), with the emission peaks of the combined systems observed at approximately 346 nm. The emission peaks of the WPC–Sop hybrid system underwent redshifts of 8 nm, 7 nm, and 6 nm at 363 K, 368 K, and 373 K, respectively. The intrinsic fluorescence quenching rate of WPC was recorded at 46.14%, 42.97%, and 51% at a Sop concentration of 200 μg/mL. The emission peaks of the WPC–inulin–Sop system exhibited a redshift of 6 nm at 363 K and 373 K, and of 7 nm at 368 K. The quenching rates at 363 K, 368 K, and 373 K were 41.82%, 39.15%, and 37.16%, respectively, and the quenching rates decreased with increasing temperature after the addition of inulin. Additionally, alterations in the amino acid microenvironment were noted following changes in Sop concentration, likely due to interactions between Sop and the WPC’s surface [39].

#### 3.2.2. Quenching Type Analysis

There are two primary mechanisms in the process of fluorescence quenching: dynamic quenching (resulting from the collision between the fluorophore and quencher) and static quenching (resulting from the formation of a ground-state complex between the fluorophore and quencher). For static quenching, quenching rate constants decrease with increasing temperature; for dynamic quenching, quenching rate constants increase with rising temperature. The quenching phenomenon in this system could be interpreted by the Stern–Volmer equation [40]. It was feasible for both quenching methods to coexist simultaneously; during this coexistence, the correlation between F_0_/F and [Q] exhibited an upward bending and concavity towards the Y-axis [41].

(1) $$F_{0}/F=1+K_{q}\tau_{0}\left[Q\right]=1+K_{SV}\left[Q\right]$$

Equation (1) describes the Stern–Volmer equation, where F is the fluorescence intensity with Sop, F_0_ is the intensity without Sop, Q is the quencher concentration, K_sv_ is the Stern–Volmer quenching rate constant, calculated by plotting F_0_/F versus Q, K_q_ is the bimolecular quenching rate constant, and τ_0_ the molecular fluorescence lifetime, which is set at 10^−8^ s [42].

**Figure 4:**
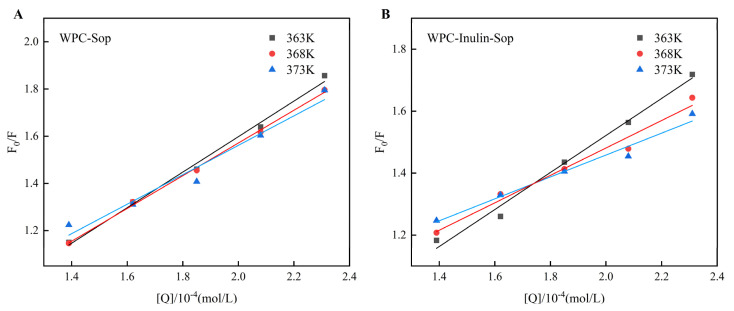
Stern-Volmer curve of interaction between Sop and WPC before and after inulin addition. (**A**) illustrates the outcomes of the SV equation calculation for the WPC-Sop system, whereas (**B**) depicts the results of the SV equation calculation for the WPC-inulin-Sop system.

A linear relationship was noted between F_0_/F and [Q] (Figure 4 and Table 1). With rising temperatures, K_sv_ decreased, and K_q_ exceeded the maximum diffusion collision quenching rate constant (2.0 × 10^10^ L·mol^−1^·s^−1^). Prior to and following inulin addition, the quenching mechanism of Sop on WPC fluorescent groups was static, indicating a stable complex formation between Sop and WPC, corroborated by UV–Vis data.

#### 3.2.3. Binding Constants and Binding Sites

The binding constant and number of binding sites reflect compound binding levels, with more sites suggesting higher constants and stronger binding. These parameters are derived using a double logarithmic equation [43].

(2) $$lg\left[\left(F_{0}-F\right)/F\right]=lgK_{a}+nlg\left[Q\right]$$

Equation (2) is a double logarithmic equation, where F represents the fluorescence intensity with Sop, F_0_ is the intensity without Sop, Q is the quencher concentration, and K_a_ and n are the binding constant and the number of binding sites, respectively.

**Figure 5:**
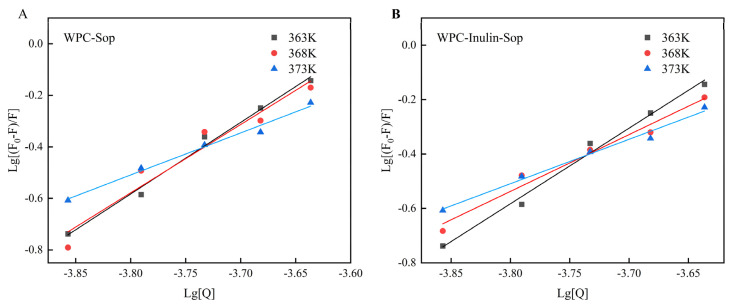
Double logarithmic curves of interaction between Sop and WPC before and after inulin addition. (**A**) illustrates the outcomes of the double logarithmic equation calculation for the WPC-Sop system. (**B**) depicts the results of the double logarithmic equation calculation for the WPC-inulin-Sop system.

As shown in Figure 5 and Table 2, incorporating inulin and increasing temperature reduced the binding affinity between Sop and WPC. Notably, the number of binding sites decreased from three to two as the temperature rose from 363 K to 368 K. At 373 K, the number of sites remained at two, with a slight decline in K_a_, possibly explaining the observed decrease in the quenching rate with increasing temperature after inulin addition. The addition of inulin at the same temperature led to reduced binding between WPC and Sop and fewer binding sites, suggesting that inulin interferes with Sop’s proximity to WPC.

#### 3.2.4. Analysis of Acting Forces

Non-covalent intermolecular forces include Van der Waals forces, hydrogen bonding, electrostatic forces, and hydrophobic interactions, identified through thermodynamic parameter calculations [44]. ΔH > 0 and ΔS > 0 indicate hydrophobic interactions, ΔH < 0 with ΔS < 0 indicates hydrogen bonding and van der Waals interactions, and ΔH < 0 and ΔS > 0 represent electrostatic interactions.


(3) $$lnK_{2}/K_{1}=\left(1/T_{1}-1/T_{2}\right)\Delta H/R$$

(4) ΔG=−RTlnK=ΔH−TΔS

In Equations (3) and (4), K_1_ and K_2_ are the binding constants at temperatures T_1_ and T_2_, respectively. ΔH, ΔS, and ΔG represent enthalpy, entropy, and Gibbs free energy changes, respectively. The universal gas constant R was 8.314 J K^−1^ mol^−1^.

For the WP–Sop system, both ΔH and ΔS are negative, suggesting that hydrogen bonding and van der Waals forces dominate the WPC–Sop interactions, and that Sop can improve the oxidation resistance of wood-plastic through this stabilizing binding energy [45]. Inulin affects only the extent of the interaction between Sop and WPC, without altering the nature of the non-covalent forces (Table 3).

#### 3.2.5. Combined Distance Analysis

A theoretical analysis and calculation of the overlapping spectra of Sop and WPC were conducted according to non-radiative energy transfer theory. This determined the binding distance and associated parameters of Sop and WPC [43].

(5) $$E=1-F/F_{0}=R_{0}^{2}/\left(R_{0}^{6}+r_{0}^{6}\right)$$

(6) $$R_{0}^{6}=8.8\times 10^{-25}K^{2}N^{-4}\Phi J$$

(7) $$J=\Sigma F\left(\lambda \right)\varepsilon \left(\lambda \right)\lambda^{4}\Delta \lambda /\Sigma F\left(\lambda \right)\Delta \lambda$$

In Equations (5)–(7), E denotes the efficiency of transfer, while R_0_ is the critical distance where E = 50%. These equations also define the distance r_0_ between the dipole centers of the energy donor and acceptor. K^2^ represents the factor determining the spatial orientation of dipoles, taking the average 2/3, N = 1.336, Φ = 0.15. J indicates the spectral overlap integral, and F(λ) and ε(λ) are the fluorescence intensity and molar absorption coefficient, respectively.

**Figure 6:**
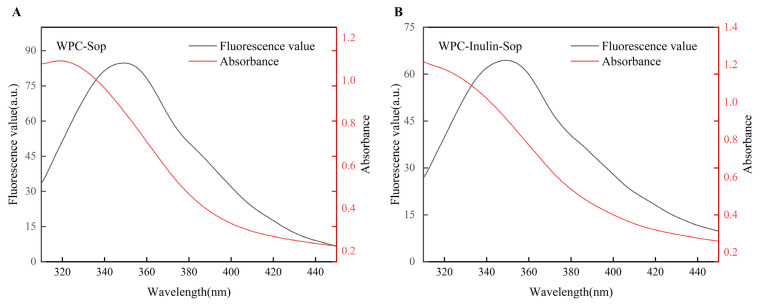
Overlapping spectra of the WPC fluorescence spectrum and Sop ultraviolet absorption spectrum. (**A**) illustrates the spectral overlap integral spectrum of the WPC–Sop system, while (**B**) depicts the spectral overlap integral spectrum of the WPC–inulin–Sop system.

At 373 K and a Sop concentration of 200 μg/mL, effective energy transfer due to significant spectral overlap between Sop and WPC was noted in Figure 6. The non-radiative energy transfer can be classified into two categories: intramolecular and intermolecular. R_0_ < 7 nm and 0.5R_0_ < R_0_ < 1.5R_0_ (Table 4), indicating energy transfer from WPC to Sop. This supports the static quenching of fluorescent groups. The addition of inulin increased the binding distance between Sop and WPC, aligning with the reduction in binding sites observed following inulin addition.

#### 3.2.6. Synchronous Fluorescence Spectrum Analysis

Shifts in the absorption peak within the synchronous spectrum indicate changes in the local environment of amino acid residues: a red shift suggests increased polarity, while a blue shift indicates increased hydrophobicity. Changes in the vicinity of the tyrosine and tryptophan residues can be highlighted by using wavelength intervals of Δλ = 15 nm and Δλ = 60 nm [46].

**Figure 7:**
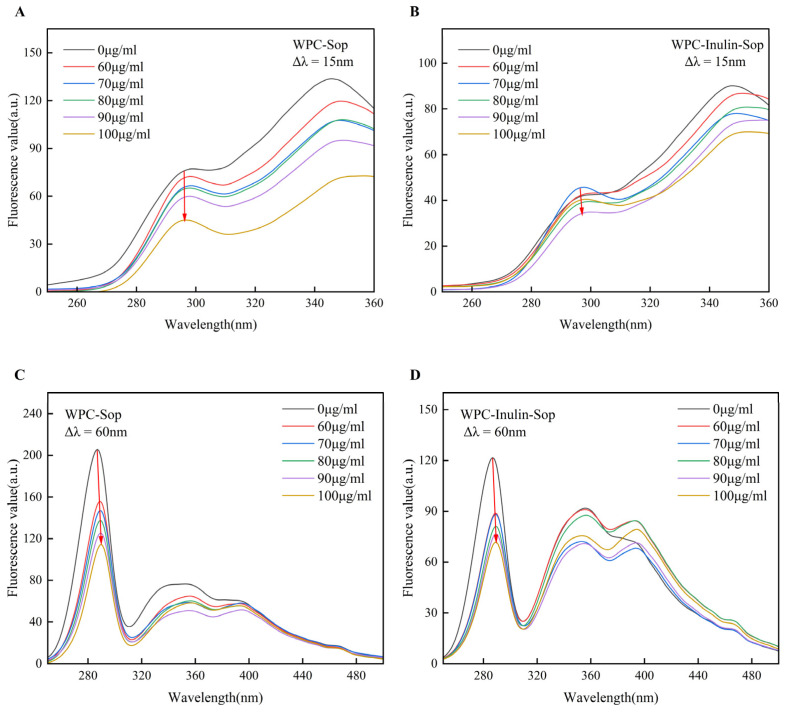
Synchronous fluorescence spectra of the interaction between Sop and WPC before and after inulin addition. (**A**,**B**) illustrate the synchronized fluorescence patterns of the WPC–Sop system and the WPC–inulin–Sop system at Δλ = 15 nm, while (**C**,**D**) depict the synchronized fluorescence patterns of the WPC–Sop system and the WPC–inulin–Sop system at Δλ = 60 nm.

The synchronous spectrum of the sample treated at 373 K was examined. Figure 7 shows that after adding Sop, the characteristic peak of tyrosine remained relatively stable, while a slight red shift was noted in the tryptophan peak. This indicates that Sop’s interaction with WPC minimally affects the microenvironment around tyrosine residues but increases the exposure of tryptophan residues to a more polar environment. In the WPC–Sop system, the fluorescence value of tyrosine decreased by 41.85% and that of tryptophan decreased by 44.57% at a Sop concentration of 200 μg/mL. In the WPC–inulin–Sop system, tyrosine fluorescence was minimally affected by Sop, decreasing by only 4.07% at 200 μg/mL, whereas tryptophan fluorescence decreased by 41.01%. Given the higher quenching rate of tryptophan compared to tyrosine, it can be concluded that tryptophan is located closer to the tyrosine site.

#### 3.2.7. Three-Dimensional Fluorescence Spectrum

**Figure 8:**
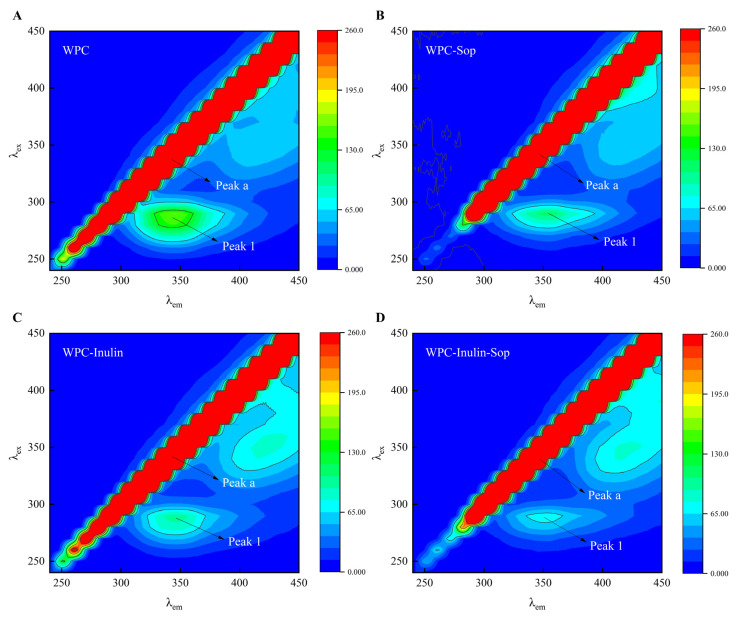
Three-dimensional spectra of the interaction between inulin, Sop, and whey protein. (**A**,**B**) illustrate the three-dimensional fluorescence spectra of the WPC–Sop system at Sop concentrations of 0 and 200 μg/mL, respectively. (**C**,**D**) present the three-dimensional fluorescence spectra of the WPC–inulin–Sop system at Sop concentrations of 0 and 200 μg/mL, respectively.

Three-dimensional fluorescence spectroscopy was used to assess structural changes in proteins. Figure 8 illustrates the interaction between Sop and WPC in the absence and presence of inulin at 373 K and Sop concentrations of 0 and 200 μg/mL. Peak 1 indicates the characteristic spectral peak of endogenous fluorescent tyrosine and tryptophan in WPC, observed at 280 nm/340 nm (λex/λem). Additionally, peak A represents the first-order Rayleigh scattering peak (λem = λex), which is affected by the degree of protein aggregation. The addition of Sop alone significantly reduced the intensity of peak 1, indicating changes in the microenvironments of tyrosine and tryptophan. This finding aligns with the synchrotron fluorescence results [47]. The introduction of inulin alone significantly weakened the intensity of peak 1, and the combined addition of Sop and inulin led to a further reduction in fluorescence intensity, accompanied by the significant redshift of peak 1.

### 3.3. FTIR Measurement

FTIR spectroscopy can characterize changes in proteins’ secondary structure and functional groups. FTIR analysis identifies alterations in proteins’ secondary structures and functional groups. The Amide I band, located at 1600–1700 cm^−1^ (Amide I band), is most commonly used to analyze proteins’ secondary structures through FTIR spectroscopy [48]. The region spanning 2800 cm^−1^ to 3100 cm^−1^ corresponds to C–H stretching vibrations associated with hydrophobic forces, while the bands between 3100–3500 cm^−1^ reflect hydrogen bond formation and changes [49].

**Figure 9:**
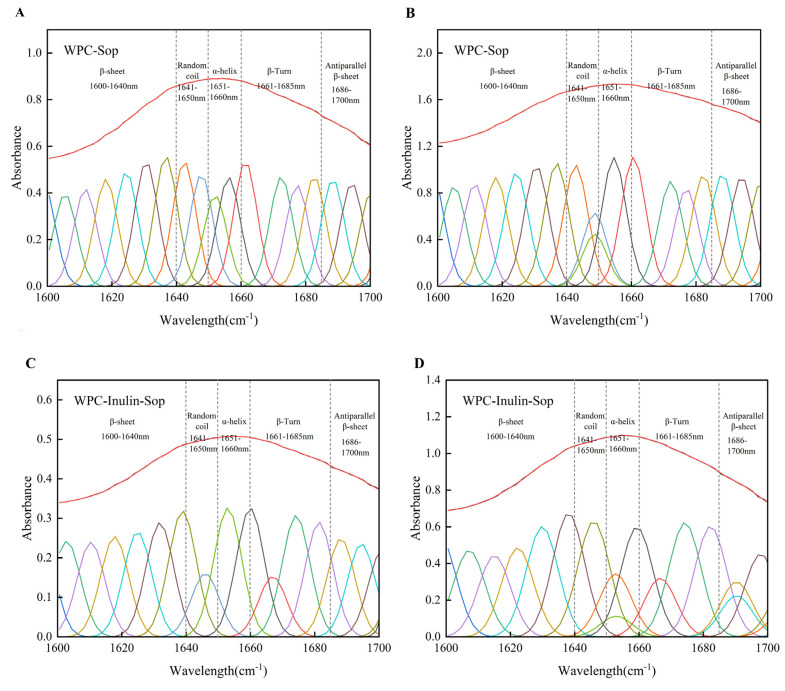
FT-IR curve fitting results of the WPC amide I band before and after inulin addition (1600–1700 cm^−1^). (**A**,**B**) illustrate the secondary structure fits of the WPC-Sop system at Sop concentrations of 0 and 200 μg/mL, respectively. (**C**,**D**) depict the secondary structure fits of the WPC-inulin-Sop system at Sop concentrations of 0 and 200 μg/mL, respectively.

Infrared data for WPC at Sop concentrations of 0 and 200 μg/mL were processed using OMNIC and Peakfitv4.12, and the results are presented in Figure 9 and Table 5. WPC’s secondary structure includes α-helixes, β-folding, antiparallel β-folding, β-turns, and irregular curling. The addition of Sop or inulin alone led to decreased α-helical and irregularly curled components and increased β-fold content. In addition, the addition of SOP decreased the number of β-turns and increased the number of antiparallel β-folds. It is noteworthy that the number of β-turns increased and the number of antiparallel β-foldings decreased when inulin was used alone. The pattern of changes when adding both inulin and Sop matched the pattern with inulin alone, suggesting inulin’s stronger effect on WPC’s secondary structure. The addition of Sop, either alone or with inulin, positively affected the development of the WPC secondary structure, enhancing orderliness. This finding suggests that adding inulin or Sop helps maintain WPC stability under heat. The β-fold was consistently the most prominent structure observed in WPC.

**Figure 10:**
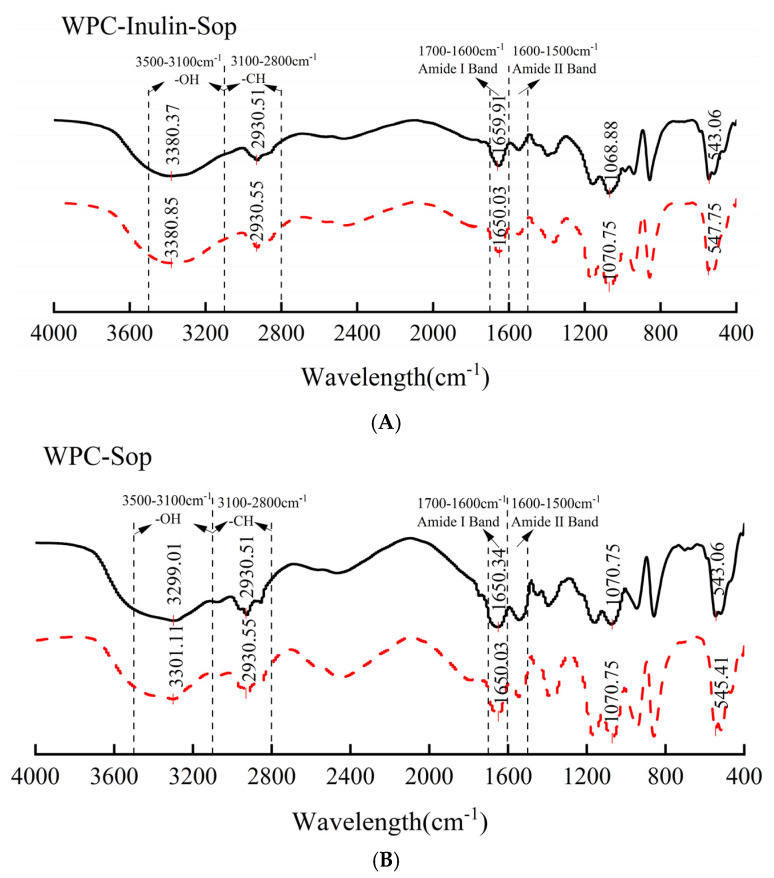
The FT-IR transmittance of WPC before and after inulin addition. (**A**) illustrates the transmittance of the WPC–Sop system, while (**B**) depicts the transmittance of the WPC–inulin–Sop system. The dashed line represents a Sop concentration of 0 μg/mL, while the solid line represents a Sop concentration of 200 μg/mL.

FTIR transmittance analysis revealed an absorption peak at approximately 2930 cm^−1^, indicating the role of hydrophobic forces in Figure 10. The band from 3500–3100 cm^−1^ showed an absorption peak at about 3300 cm^−1^, with a significant red shift after adding inulin, implying hydrogen bond involvement in compound interactions, consistent with thermodynamic findings. Additional characteristic peaks at 1650 cm^−1^, 1070 cm^−1^, and 545 cm^−1^ corresponded to C-O stretching, C-H stretching, and aromatic hydrocarbons’ plane bending in the amide I band, respectively [33].

### 3.4. Molecular Docking Simulations

The results presented in Table 6, demonstrated interactions between Sop and various WPC proteins. Key hydrogen bonding amino acids with Sop included ASN-44 (2.3 Å), 45-ASN (2.0 Å), 102-ASN (2.2 Å), 101-ILE (2.4 Å; 2.5 Å), and ASP-97 (2.2 Å) in α-La; Lys-70 (2.1 Å; 2.3 Å), Ile-72 (1.9 Å; 2.7 Å), and Glu-74 (1.9 Å) in β-Lg, and Asp-85 (2.5 Å), Glu-125 (2.6 Å) and Lys-136 (2.5 Å; 2.6 Å) in BSA; Arg-309 (2.2 Å; 3.1 Å), Glu-682 (2.6 Å), and Ala-689 (2.7 Å) in LF. These results align with previous analyses emphasizing hydrogen bonding’s critical role in Sop–protein interactions.

Table 7 shows the docking results for Sop and WPC proteins. Notably, α-La exhibited the lowest docking energy at −5.57 kcal/mol, and LF exhibited the highest at −4.43 kcal/mol. Sop’s docking energy with each protein was consistently below −1.2 kcal/mol, indicating effective interactions between Sop and WPC.

### 3.5. Surface Hydrophobicity

**Figure 11:**
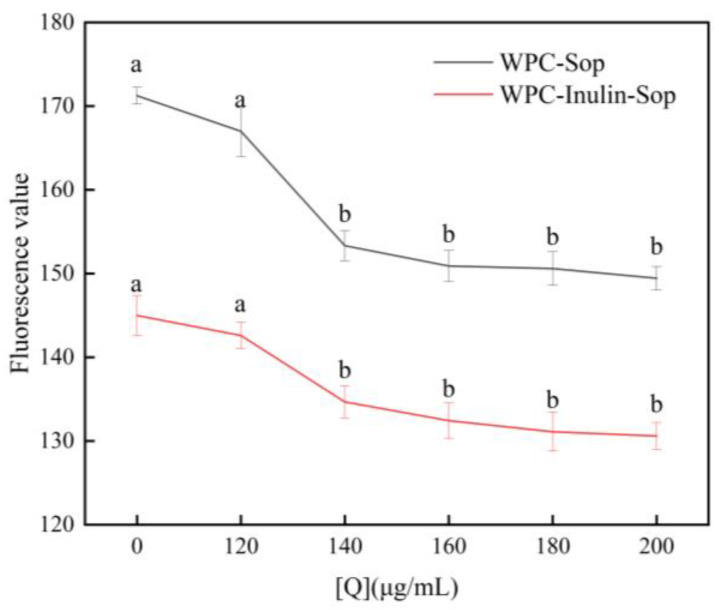
Effect of the Sop concentration change before and after inulin addition on the surface hydrophobicity of WPC. Note: Different letters in the same row indicate significant differences (*p* < 0.05).

The intrinsic fluorescence intensity of ANS was weak; however, binding to hydrophobic amino acids on the protein surface markedly increased the intensity [50]. As illustrated in Figure 11 and Table 8, the hydrophobicity of WPC decreased with the increase in Sop concentration, and, after the addition of inulin, the hydrophobicity was further decreased, which may be due to the exposure of hydrophilic groups. Enhanced hydrophilicity improves protein solubility and is crucial for enhancing protein emulsification, particularly under weakly alkaline conditions [51].

## 4. Conclusions

The effect of inulin on the interaction between WPC and Sop was examined using various analytical methods, including UV–Vis spectroscopy, fluorescence spectroscopy, FTIR spectroscopy, molecular docking, and surface hydrophobicity analysis. It was observed that Sop and WPC could spontaneously associate to form a complex, potentially enhancing the antioxidant properties of WPC. However, inulin appeared to inhibit this complex formation. The incorporation of either Sop or inulin, individually or combined, augmented the ordering of WPC’s secondary structure, suggesting that both agents could enhance the protein’s thermal stability. Surface hydrophobicity of WPC, assessed via the ANS fluorescence probe method, revealed that both Sop and inulin reduced WPC’s hydrophobicity. Consequently, they increased WPC’s solubility and potentially improved its emulsification properties.

## Figures and Tables

**Table 1:** Fluorescence quenching parameters of interaction between Sop and WPC before and after inulin addition.

System	T/K	K_sv_ (10^4^ L·mol^−1^)	K_q_ (10^12^ L·mol^−1^·s^−1^)	R^2^
WPC-Sop	363	0.75	0.75	0.99
	368	0.66	0.66	0.99
	373	0.62	0.62	0.96
WPC-inulin-Sop	363	0.60	0.60	0.99
	368	0.44	0.44	0.97
	373	0.35	0.35	0.98

**Table 2:** Binding parameters of interaction between Sop and WPC before and after inulin addition.

System	T/K	K_a_/L·mol^−1^	N	R^2^
WPC-Sop	363	1.15 × 10^12^	3.31	0.98
	368	3.16 × 10^9^	2.65	0.95
	373	1.05 × 10^9^	2.51	0.99
WPC-inulin-Sop	363	9.55 × 10^9^	2.76	0.99
	368	2.51 × 10^7^	2.09	0.98
	373	5.01 × 10^5^	1.62	0.99

**Table 3:** Thermodynamic parameters of the interaction between Sop and WPC before and after inulin addition.

System	T/K	ΔH (KJ·mol^−1^)	ΔS (KJ·mol^−1^·K^−1^)	ΔG (KJ·mol^−1^)
WPC-Sop	363	−787.98	−1.94	−83.81
	368			−74.06
	373			−64.41
WPC-inulin-Sop	363	−1109.39	−2.87	−69.35
	368			−53.23
	373			−40.70

**Table 4:** Binding distance between Sop and WPC before and after inulin addition.

System	Sop (mol/mL)	E	J (10^−15^ L·cm^−3^·mol^−1^)	R_0_/nm	r_0_/nm
WPC-Sop	0.231	0.44	4.815.25	2.11	2.19
WPC-inulin-Sop		0.37		2.14	2.34

**Table 5:** Effect of Sop on the content change in secondary structure of WPC before and after inulin addition.

System	Sop (μg/mL)	α-Helix (%)	β-Sheet (%)	β-Turn (%)	Antiparallel β-Sheet (%)	Random Coil (%)
WPC-Sop	0	15.93%	38.44%	22.49%	13.56%	9.58%
	200	14.91%	40.60%	21.65%	14.60%	8.25%
WPC-inulin-Sop	0	15.83%	42.74%	23.93%	9.79%	7.71%
	200	14.84%	40.79%	23.48%	12.42%	7.86%

**Table 6:** Simulation of docking between WPC protein components and Sop. (**A**-**D**) α-La; β-Lg; LF; simulation of BSA protein molecular docking.

Protein 3D Models	Detailed Surface Model
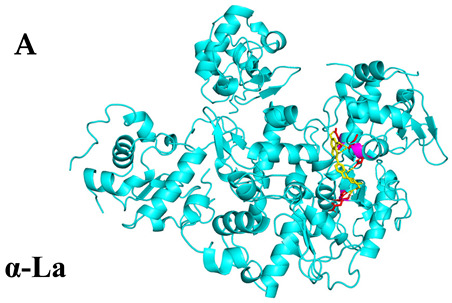	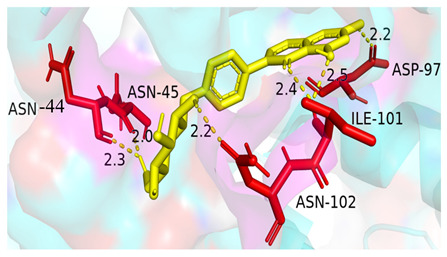
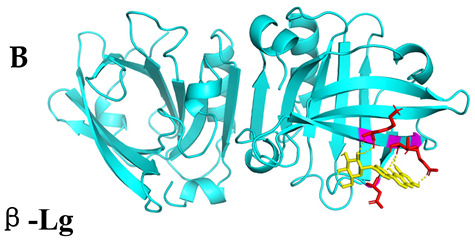	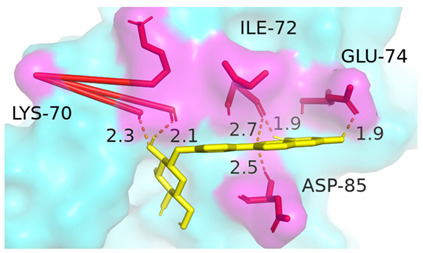
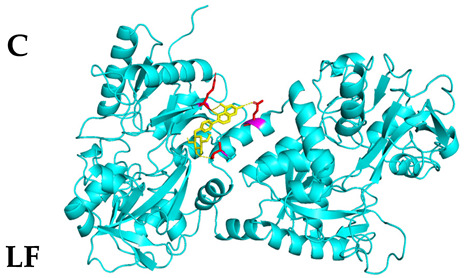	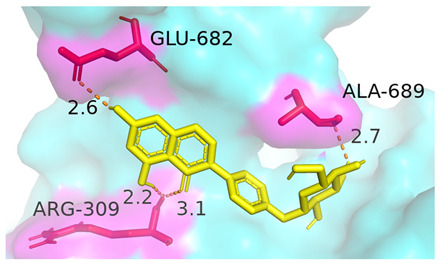
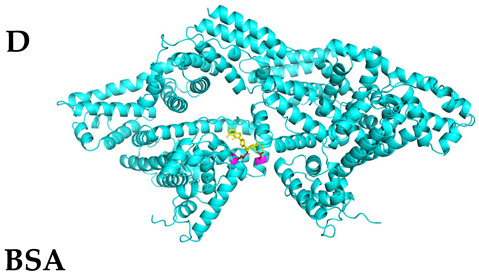	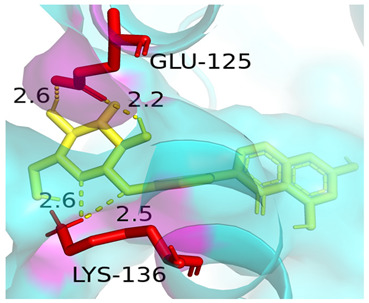

**Table 7:** Docking scores of whey protein components with Sop molecules.

Receptor	Ligand	Docking Score/(kcal·mol^−1^)
α-La	Sop	−5.57
β-Lg		−4.67
BSA		−4.63
LF		−4.43

**Table 8:** Effect of the Sop concentration change before and after inulin addition on the surface hydrophobicity of WPC.

	*Sop* Conc. (μg/mL)	0	120	140	160	180	200
System							
WPC-Sop		171.23 ± 1.00 ^a^	167.00 ± 3.04 ^a^	153.33 ± 1.80 ^b^	150.90 ± 1.85 ^b^	150.60 ± 2.00 ^b^	149.43 ± 1.40 ^b^
WPC-inulin-Sop		145.00 ± 2.39 ^a^	142.60 ± 1.55 ^a^	134.67 ± 1.92 ^b^	132.43 ± 2.11 ^b^	131.10 ± 2.31 ^b^	130.60 ± 1.61 ^b^

Note: Different letters in the same row indicate significant differences (*p* < 0.05).

## Data Availability

The original contributions presented in the study are included in the article, further inquiries can be directed to the corresponding author.
